# Response to post-axitinib treatment in patients with metastatic renal cell carcinoma

**DOI:** 10.1186/s12885-016-2282-5

**Published:** 2016-03-29

**Authors:** Namita Chittoria, Housam Haddad, Paul Elson, Nizar M. Tannir, Laura S. Wood, Robert Dreicer, Jorge A. Garcia, Brian I. Rini, Eric Jonasch

**Affiliations:** Cleveland Clinic Taussig Cancer Center, 9500 Euclid Ave., Cleveland, OH USA; University of Texas M. D. Anderson Cancer Center, Houston, TX USA; University of Virginia, 1240 Lee St., Charlottesville, VA USA; Veteran Affairs Medical Center, 800 Irving Avenue, Syracuse, NY 13210 USA

**Keywords:** Renal cell carcinoma, Axitinib, Sunitinib, VEGF inhibitors, mTOR inhibitors, Predictive biomarkers

## Abstract

**Background:**

Axitinib is a potent inhibitor of the vascular endothelial growth factor (VEGF) receptor family with clinical activity in patients with metastatic renal cell carcinoma (mRCC). Given this biochemical potency, the clinical activity of subsequent treatment with targeted therapies in patients progressing on axitinib is of interest.

**Methods:**

Patients with advanced renal cell carcinoma of any pathologic subtype treated with at least one cycle (four weeks) of axitinib followed by at least one subsequent targeted therapy were investigated in a retrospective analysis. Patient characteristics, duration of treatment and clinical outcomes were analyzed for axitinib and each subsequent line of therapy by Response Evaluation Criteria in Solid Tumors (RECIST).

**Results:**

Twenty-five mRCC patients who received at least one approved targeted agent following axitinib were identified. Eight percent of patients achieved a partial response (one patient each to sunitinib and pazopanib) and 42 % had a best response of stable disease to the first therapy after axitinib. The estimated median duration of therapy was 4.4 months (range, 0.2–27.5+). Twelve patients received a second post-axitinib targeted therapy. Six out of 11 evaluable patients (55 %) had a best response of SD. The estimated median duration of treatment was 4.8 months (range, 0.7–19.1+).

**Conclusion:**

Objective responses and stable disease is observed to post-axitinib targeted therapies and prospective studies are needed for validating role of predictive biomarkers.

## Background

Renal cell carcinoma (RCC) is a biologically heterogeneous disease with distinct genetic and metabolic defects [1]. Over the past decade, recognition that von Hippel-Lindau (*VHL*) gene mutations cause overexpression of vascular endothelial growth factor (VEGF) and increased tumor angiogenesis has led to development of multiple agents targeting this protein and its receptor.

Currently approved therapies for treatment of patients with mRCC include bevacizumab (plus interferon alfa), a humanized monoclonal antibody that inhibits the VEGF ligand, and the multi-targeted receptor tyrosine kinase inhibitors, sunitinib, sorafenib, pazopanib and axitinib (VEGFr- TKIs) [2–7]. Each agent has a slightly different affinity for the VEGF and platelet derived growth factor (PDGF) receptors, as well as for other receptor tyrosine kinases [8]. Mammalian target of rapamycin (mTOR) inhibitors, which include everolimus and temsirolimus [9, 10] are also approved for treatment of mRCC, and do not appear to have a direct effect on VEGF or its receptors.

The most recently Food and Drug Administration (FDA) approved agent for mRCC is axitinib, a second-generation, indazole derived molecule that binds selectively to the adenosine triphosphate (ATP)-binding intracellular domain of VEGFR-1, 2, and 3 at sub-nanomolar concentrations. The AXIS trial that led to the approval of axitinib was a phase 3, randomized controlled study comparing two VEGFr TKIs, axitinib and sorafenib, in patients whose disease progressed on initial systemic therapy [7]. Patients in each treatment arm had received first-line treatment with sunitinib (54 %), cytokines (35 %), bevacizumab (8 %), or temsirolimus (3 %). In the overall population, patients treated with axitinib experienced a significantly longer median progression free survival (PFS) than patients treated with sorafenib (6.7 vs. 4.7 months; *P* < 0.0001). Secondary endpoints included overall response rate (ORR), overall survival (OS), and safety and tolerability. ORR was 19.4 % (95 % Cl 15.4–23.9 %) versus 9.4 % (95 % CI 6.6–12.9 %) for axitinib and sorafenib, respectively. In the sub-group of sunitinib-refractory patients, median PFS was 4.8 months for patients treated with second-line axitinib and 3.4 months for patients treated with second-line sorafenib (*P* = 0.0107). In the subgroup of cytokine-refractory patients, median PFS was 12.1 months for patients treated with second-line axitinib and 6.5months for patients treated with second-line sorafenib (*P* < 0.0001) [11]. The longer median PFS values observed in cytokine-refractory patients relative to sunitinib-refractory patients points to partial cross-resistance with sequential VEGF-targeted therapy [11]. This suggests that targeting of the same pathway with sequential VEGFr-TKI therapy may follow a law of diminishing returns due to unknown mechanisms of increasing resistance [12].

Knowing that axitinib is the most biochemically potent of the approved VEGFr inhibitors, and that there is possibility of cross-resistance with sequential VEGF-targeting therapy, the response to therapy after progressing on axitinib is of clinical interest.

A retrospective review of patients from the Cleveland Clinic Taussig Cancer Center (CCF) and MD Anderson Cancer Center (MDACC) was thus undertaken to characterize and evaluate the response to subsequent systemic therapy in patients who had progressed on axitinib.

## Methods

### Study design and patient characteristics

Patients from CCF or MDACC were identified through prospectively maintained databases and included in the study, which was approved by Cleveland Clinic and UT MD Anderson institutional review board. The initial criteria for case identification included a diagnosis of metastatic RCC of any pathologic subtype and treatment with at least one cycle (four weeks) of axitinib. Eighty-one patients treated with axitinib between November 2003 and August 2010 were initially identified; 25 patients (17 (68 %) from CCF and 8 (32 %) from MDACC) received at least one approved targeted agent following axitinib and were included in this analysis. The remaining patients were excluded due to ≤ 4 weeks on axitinib (*n* = 3), ongoing axitinib therapy (*n* = 16), lack of subsequent systemic therapy (*n* = 13), lost to follow up (*n* = 11), receiving non targeted or investigational agents post-axitinib (*n* = 11) or were not evaluable (*n* = 2). All patients were enrolled in axitinib clinical trials given that axitinib therapy pre-dated FDA approval.

Data collection was performed via retrospective review of each patient’s medical record and recorded on a spreadsheet standardized between the two centers. Patient characteristics, duration of treatment and clinical outcomes were analyzed for axitinib and each subsequent line of therapy. Objective response was defined according to RECIST version 1.0. All imaging studies were done at MDACC or CCF and response was evaluated by treating physicians at each institute. There was no centralized review of the radiological findings. Response beyond the second subsequent therapy was not evaluated because of lack of sufficient data.

### Statistical analyses

Categorical data were summarized as frequency counts and percentages and measured data by medians and ranges. The Kaplan Meier method was used to summarize the duration of subsequent treatments since some patients were still receiving therapy at the time of analysis. The Wilcoxon signed rank test was used to compare treatment duration with axitinib to the duration on subsequent therapy in patients with complete data. Data analysis was conducted using SAS version 9.2 (SAS Inc., Cary NC)

## Results

### Patient characteristics

Twenty-five patients (17 (68 %) from CCF and 8 (32 %) from MDACC) received at least one approved targeted agent following axitinib and were included in this analysis. Patient characteristics at the start of axitinib were typical of an advanced RCC population and included 72 % male, median age 59 (range, 44–78); 96 % clear cell; 92 % prior nephrectomy; 72 % previously-treated. Patients had favorable (30 %) or intermediate (65 %) risk disease based on Heng criteria [13]. The overall RECIST-defined objective response rate to axitinib was 56 % (one complete and 13 partial response) and the median duration of treatment with axitinib was 11.2 months (range, 1.1–90) (Table 1).

**Table 1:** Patient characteristics and clinical outcomes to axitinib

Characteristic	N (%)
Gender	
Male	18 (72 %)
Female	7 (28 %)
Age at Start of Axitinib (years)^a^	
Median (range)	59 (44-78)
Histology	
Clear cell	24 (96 %)
Unclassified	1 (4 %)
Prior Nephrectomy	
No	2 (8 %)
Yes	23 (92 %)
Prior Systemic Treatment	
No	7 (28 %)
Yes	18 (72 %)
IFN and/or IL-2	11 (44 %)
Sorafenib	9 (36 %)
Sunitinib	6 (24 %)
Temsirolimus	1 (4 %)
Bevacizumab	1 (3 %)
Other^b^	4 (16 %)
Interval from Dx of Mets to Axitinib^a^	
Median in months (range)	20.1 (0.2–49.9)
ECOG PS	
0	7 (28 %)
1	18 (72 %)
Heng Risk Group^c^	
Favorable	7 (30 %)
Intermediate	15 (65 %)
Unfavorable	1 (4 %)
Sites of Metastatic Disease	
Lung	20 (80 %)
Lymph nodes	13 (52 %)
Bone	6 (24 %)
Liver	5 (20 %)
Adrenal	5 (20 %)
Pancreas	4 (16 %)
Brain	2 (8 %)
Other^d^	12 (48 %)
Best Response to axitinib	
CR	1 (4 %)
PR	13 (52 %)
SD	10 (40 %)
PD	1 (4 %)
Reason Axitinib Stopped	
PD	21 (84 %)
Toxicity	4 (16 %)
Duration of Treatment	
Median in months (range)	11.2 (1.1–90.0)

^a^missing for one patient

^b^alone or in combination: thalidomide (*n* = 3); gemcitabine,5-FU, ABX-EGF, capecitabine, lenalidomide, suramin, vinblastine (*n* = 1 each)

^c^missing for two patients

^d^kidney/renal bed (*n* = 5); pleura (*n* = 4); abdominal wall, muscle, omentum, pelvic mass, retroperitoneum, soft tissue (*n* = 1 each)

### Response to first subsequent therapy post axitinib

Following axitinib therapy, patients were treated with VEGF receptor inhibitors (*n* = 18) or mTOR inhibitors (*n* = 7). Overall, 8 % of patients achieved a partial response (one patient each to sunitinib and pazopanib) and 42 % had a best response of stable disease (nine patients to VEGF receptor inhibitors and four patients to mTOR inhibitors). The estimated median duration of subsequent therapy was 4.4 months (range, 0.2–27.5+) (Table 2).

**Table 2:** Patient characteristics and clinical outcomes to post-axitinib systemic therapy

Factor		First post-axitinib therapy (*n* = 25)	Second post-axitinib therapy (*n* = 12)
Interval from End of Axitinib Therapy to Start of Current Therapy Median in weeks (range)		2.0 (0–41.7)^a^	37.4 (2.6–93.0)
Treatment		N ( %)	N ( %)
VEGF inhibitors		18 (72 %)	3 (25 %)
Sunitinib		8 (32 %)	−0-
Pazopanib		6 (24 %)	1 (8 %)
Bevacizumab		4 (16 %)	2 (17 %)
mTOR inhibitors		7 (28 %)	8 (67 %)
Everolimus		5 (20 %)	5 (42 %)
Temsirolimus		2 (8 %)	3 (25 %)
Other		−0-	1 (8 %)^b^
ECOG PS			
0		3 (13 %)	2 (17 %)
1		19 (79 %)	9 (75 %)
2		2 (8 %)^a^	1 (8 %)
Heng Risk Group			
Favorable		9 (41 %)	3 (30 %)
Intermediate		12 (55 %)	6 (60 %)
Unfavorable		1 (5 %)^c^	1 (10 %)^d^
Best Response			
PD		2 (8 %)	−0-
SD		13 (42 %)	6 (55 %)
PD		7 (28 %)	4 (36 %)
Not evaluable		3 (12 %)	1 (9 %)^a^
Reason Treatment Stopped			
PD/Death		17 (68 %)	6 (54 %)
Toxicity		4 (16 %)	2 (18 %)
Treatment ongoing		4 (16 %)	3 (27 %)
Duration of Treatment (subsequent therapy) Median in months (range)		4.4 (0.2–27.5+)^a^	4.8 (0.7–19.1+)^a^

^a^missing for one patient

^b^MK2206

^c^missing for three patients

^d^missing for two patients

The response to first subsequent therapy was evaluated in the subgroup of patients who had received axitinib as second-line or later therapy (72 %, 18/25) and in patients who had received no therapy prior to axitinib (28 %, 7/25). Ten patients (55 %) had a best response of stable disease in the group of patients who had received axitinib as second-line or later therapy as compared to three (42 %) in the latter group. One patient achieved partial response in both groups (Table 3).

**Table 3:** Prior treatments and clinical outcomes to axitinib and first subsequent therapy

Patient ID	Therapies prior to Axitinib	Duration of axitinib treatment (mon)	Best response to Axitinib	Reason for discontinuation	Interval from End of Axitinib Therapy to Start of Subsequent Therapy (weeks)	Subsequent therapy 1	Best response
1	IL-2 plus IFN	18	PR	PD	28	Sunitinib	PR
2	IL-2 plus IFN	90	PR	PD	1	Bevacizumab	SD
3	IL-2 plus IFN	24	SD	PD	41	Sunitinib	Not evaluable
4	IL-2	6	PR	PD	0	Pazopanib	PD
5	IL-2 plus thalidomide, Sorafenib	41	PR	PD	2	Sunitinib	SD
6	IL-2/Bevacizumab	18	SD	PD	3	Pazopanib	SD
7	IL-2, sunitinib, IFN, sorafenib	8	SD	PD	1	Bevacizumab	PD
8	IL-2 plus IFN, BAY 43-9006, Sorafenib	31	PR	Toxicity, RPLS^a^	10	Temserolimus	SD
9	IFN,vinblastin plus thalidomide,IL-2, Gemcitabine plus Capecitabine, Sorafenib	53	PR	Toxicity, MI^b^	7	Pazopanib	SD
10	IFN, Sunitinib, Sorafinib	8	SD	PD	35	Temserolimus	SD
11	IFN, IL-2/thalidomide, IL-2/IFN, sunitinib, sorafenib	11	SD	PD	2	Bevacizumab	PD
12	ABX-EGF, CC-5013, 5FU/suramin, Sorafinib	4	SD	PD	5	Sunitinib	SD
13	Sunitinib	1	PD	PD	0	Everolimus	Not evaluable
14	Sunitinb	3	SD	PD	1	Everolimus	PD
15	Sunitinib	3	SD	PD	0	Everolimus	SD
16	Sorafenib	19	PR	PD	2	Sunitinib	PD
17	Sorafenibb	41	PR	PD	2	Sunitinib	SD
18	Temsirolimus	6	PR	Toxicity	0	Pazopanib	SD
19	None	29	PR	PD	3	Everolimus	SD
20	None	20	PR	PD	2	Everolimus	PD
21	None	2	CR	Toxicity	0	Pazopanib	PR
22	None	4	SD	PD	32	Sunitinib	SD
23	None	13	PR	PD	2	Bevacizumab	PD
24	None	6	SD	PD	2	Pazopanib	SD
25	None	6	PR	PD	3	Sunitinib	Not evaluable

^a^Reversible Posterior Leukoencephalopathy Syndrome

^b^Myocardial Infarction

### Response to second subsequent therapy post axitinib

Eleven of twelve patients who received a second post-axitinib targeted therapy were treated with VEGF receptor inhibitors (*n* = 3) or mTOR inhibitors (*n* = 8). No partial responses were observed. However, six of 11 evaluable patients (55 %) had a best response of SD to mTOR inhibitors. The estimated median duration of treatment was 4.8 months (range, 0.7–19.1+).

Patient response to subsequent therapy was generally less favorable than the response to axitinib. This was true also for four patients who discontinued axitinib due to toxicity and not due to progressive disease. Among 13 evaluable patients who achieved a CR or PR on axitinib, only 2 (15 %) achieved a PR on their next systemic therapy, 7 (54 %) had a best response of stable disease, and 4 (31 %) progressed.

**Fig. 1:**
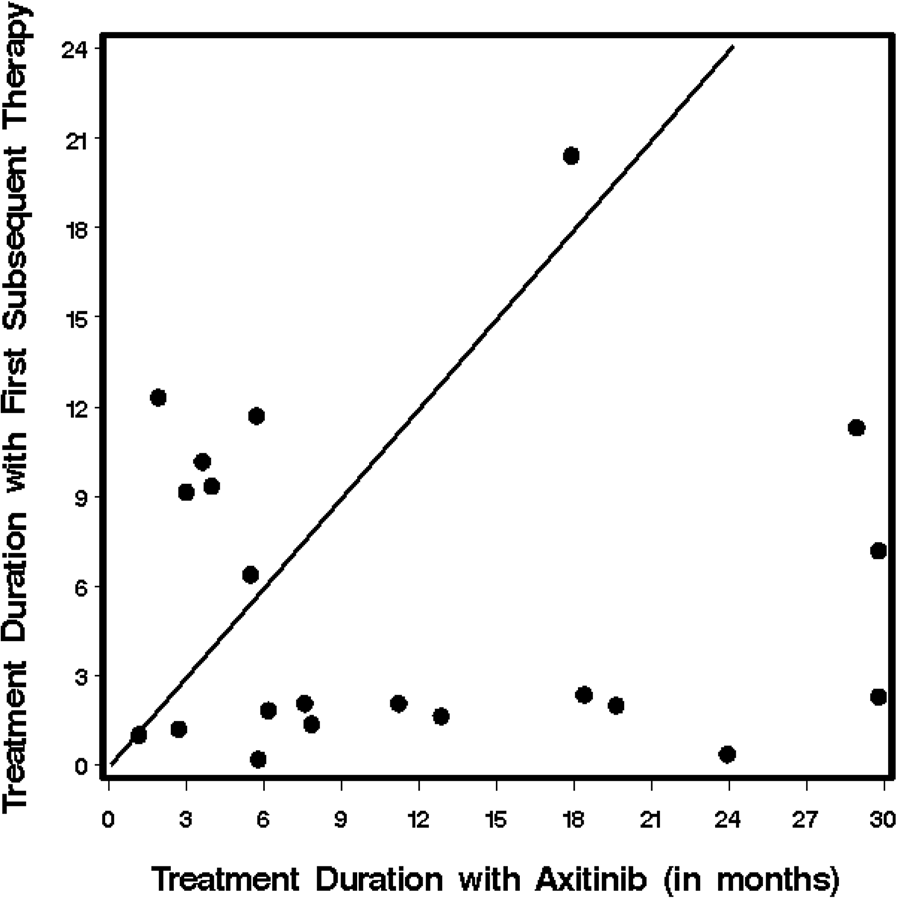
Treatment Duration with Axitinib vs First Subsequent (Post – Axitinib) Systemic Therapy. Notes: Axitinib duration cropped at 30 months for two patients (actual durations were 53.1 and 90.0 months). Data points above the diagonal represent patients who remained on treatment longer with subsequent therapy than with axitinib; points below the diagonal represent the converse

Further, Fig. 1 delineates two populations The majority of the 14 patients who had a prolonged response to axitinib had brief responses (<3 months) to subsequent therapies; however seven patients (28 %) remained on their first post-axitinib therapy longer than on axitinib. Despite this, the overall duration of axitinib treatment was longer as compared to subsequent therapy (median 11.2 versus 4.4 months, *p* = 0.04). All of these seven patients had clear cell RCC without sarcomatoid features and six of these seven patients received sunitinib or pazopanib as first subsequent therapy and only one received temsirolimus.

## Discussion

The standard of care in metastatic RCC is the empiric and sequential use of systemic therapies, most of which target VEGF. Previous retrospective and prospective evidence points to lack of complete cross-resistance to sequential VEGF-targeting therapies [14, 15], although in general the clinical activity decreases with drugs given later in the sequence. The reasons for development of resistance to initial therapy and factors affecting response to subsequent therapy are not well understood at present. The present analysis demonstrates that clinical responses (objective partial responses and stable disease) are possible if VEGF receptor inhibitors are given after axitinib, although in general the activity of subsequent therapy is less than that observed with axitinib.

It has been hypothesized that residual VEGF signaling after VEGF-targeted therapy could account for the activity of VEGF-targeted agents in this setting. Given the increased biochemical potency of axitinib, it was hypothesized that therapy (specifically VEGF-targeted therapy) after axitinib treatment may not result in clinical benefit. Our retrospective data suggests that clinical effect is seen in patients with metastatic RCC who receive systemic therapy after axitinib, not only as second but also as third line. Two thirds of our patients had received systemic therapy prior to axitinib and we still observed objective responses on subsequent therapies, median duration of therapy was over four months and seven patients remained on first subsequent therapy longer than axitinib. This may be explained by the therapeutic effect of different target engagement by the various VEGF multikinase inhibitors [16, 17]. Our study also delineates two populations, one that responds better to axitinib and another to subsequent therapy. This variable response emphasizes possible role of molecular biomarkers in guiding individualized patient management and improving outcomes.

There are limitations to this analysis. This is a retrospective review of a small number of patients at two specialized institutions with inability to overcome selection bias. Individual treating physicians at each institute did the tumor assessment, thus limiting the reliability and consistency of the percent changes in tumor burden and the assessment of objective response.

Further prospective trials are evaluating second-line treatment with approved or investigational agents in patients with mRCC who were refractory to previous treatment with a targeted agent. Other trials are evaluating the optimal sequence of targeted agents in treatment-naive patients with mRCC. Results of these trials may give us some insight into efficacy of sequenced VEGFR – TKIs and cross – resistance in metastatic renal cell carcinoma.

## Conclusion

Objective responses and stable disease is observed with post-axitinib targeted therapy, although efficacy is generally less than that seen with axitinib. Prospective studies will help us understand if prior response or resistance to axitinib predicts for clinical benefit to subsequent therapy. Variable patient response to drugs in the same class warrants prospective studies to validate role of predictive biomarkers in guiding individualized therapies and improving outcomes.

### Ethics approval and consent to participate

Waived.

### Consent for publication

Non-applicable.

### Availability of data and materials

The dataset supporting the conclusions of this article is available at request from the corresponding author.

## Abbreviations

MI: Myocardial Infarction
mRCC: metastatic renal cell carcinoma
ORR: overall response rate
OS: overall survival
PDGF: platelet derived growth factor
PFS: progression free survival
RCC: renal cell carcinoma
RECIST: response evaluation criteria in solid tumors
RPLS: Reversible Posterior Leukoencephalopathy Syndrome
VEGF: vascular endothelial growth factor
VHL: von Hippel-Lindau
